# Supplementary material for: Antimicrobial Activity of Spanish Propolis against Listeria monocytogenes and Other Listeria Strains
Source: Microorganisms. 2023 May 29;11(6):1429. doi: 10.3390/microorganisms11061429 (PMC10305080; doi:10.3390/microorganisms11061429)
Supplement: Supplementary file 1 [file microorganisms-11-01429-s001.zip › microorganisms-2398241-supplementary.pdf]

Table S1 Geographical location of the propolis samples and identification code.

Propolis	City/Region	Zone Code
**1**	Omaña	I
**2**	Valduerna	I
**3**	Matallana De Torio	I
**4**	Tafalla	III
**5**	Xinzo De Lima	V
**6**	Degaña	I
**7**	Carballiño	V
**8**	Boimorto	V
**9**	Ourense1	V
**10**	Ourense2	V
**11**	Olot	II
**12**	Mutilva	III
**13**	Vegacervera	I
**14**	Mazon	I
**15**	Olmillos De Castro	I
**16**	Ourense3	V
**17**	Ourense4	V
**18**	Valdespina	I
**19**	Mostoles	I
**20**	San Quirce Del Valles (Barcelona)	II
**21**	Ferreras 1	I
**22**	Ferreras 2	I
**23**	Secarejo 1	I
**24**	Sueros De Cepeda1	I
**25**	Ferreras3	I
**26**	Ferreras4	I

**27**	Secarejo2	I
**28**	Brañuelas1	I
**29**	Brañuelas2	I
**30**	Brañuelas3	I
**31**	Sueros De Cepeda2	I

Table S2 Physicochemical characterization of propolis samples.

Region Code	***Wax (% w/w)***	***Resin (% w/w)***	***Ash (% w/w)***	***Impurities (% w/w)***	***Water+ volatil compounds and others (% w/w)***
I	27.82-10.64	76.26-50.73	2.92-0.33	10.03-3.29	22.29-5.43
II	14.83-13.74	73.79-66.86	1.32-0.44	11.07-3.25	9.04-5.99
III	21.13-8.68	83.52-65.73	0.92-0.58	5.89-5.35	7.21-1.23
IV	27.90-7.74	71.63-48.20	0.79-0.60	15.12-4.11	12.94-6.96

Table S3 MIC and MBC values for the all the EEPs after 48 h of EEP against the strains of Listeria grayii, Listeria innocua and Listeria monocytogenes under study on three different pH conditions.

**EEP**	**PH**	***Lm 10***		***L. innocua30***		***Lm51***		***Lm52***		***Lm74***		***Lm75***		***L. innocua 910***		***Lgrayii 931***		***Lm934***		***Lm935***		***Lm4032***	
		MIC	MBC	MIC	MBC	MIC	MBC	MIC	MBC	MIC	MBC	MIC	MBC	MIC	MBC	MIC	MBC	MIC	MBC	MIC	MBC	MIC	MBC
1	7.04	4	4	1	1	1	1	1	*	1	7	2	2	2	2	1	4	4	4	2	4	2	4
	6.01	2	2	2	2	3	4	2	2	2	7	2	2	2	1	2	2	2	2	2	2	2	4
	5.01	1	1	1	1	2	1	1	1	1	2	1	1	2	1	2	1	2	1	2	1	2	1
2	7.04	4	4	4	4	4	4	1	7	4	4	2	4	4	4	2	4	4	7	2	4	4	4
	6.01	2	2	4	4	4	4	4	4	4	4	4	4	2	2	2	2	2	4	4	2	3	2
	5.01	2	2	7	2	1	1	1	2	1	2	2	2	2	2	2	2	2	2	2	2	2	2
3	7.04	2	2	2	4	4	*	2	4	4	4	4	4	2	2	2	2	1	1	4	2	4	2
	6.01	1	4	2	2	2	7	2	1	2	2	4	2	2	1	2	1	3	1	2	1	4	2
	5.01	1	2	1	1	1	1	1	1	2	2	2	1	2	1	2	1	2	1	2	1	2	1
4	7.04	2	4	2	4	4	4	4	4	4	4	2	4	2	4	1	2	2	4	2	2	2	2
	6.01	3	4	2	4	2	7	2	7	2	4	2	7	4	4	3	2	4	4	4	4	4	4
	5.01	2	1	2	2	2	1	2	1	4	2	2	2	1	2	1	1	1	2	1	1	1	1
5	7.04	1	1	1	2	2	2	1	4	1	4	2	4	1	7	2	4	1	7	1	2	1	4
	6.01	2	1	2	4	2	2	2	2	2	4	2	4	2	2	2	**	2	4	2	4	2	2
	5.01	1	1	1	1	1	1	2	1	2	2	2	4	1	4	1	1	2	1	2	1	1	1
6	7.04	3	7	4	7	1	4	4	4	2	4	4	2	5	7	2	1	4	4	2	4	2	7
	6.01	2	2	2	2	2	4	2	4	4	4	2	7	2	2	2	2	2	2	4	2	4	4
	5.01	2	2	1	1	1	1	2	1	1	2	1	2	2	1	2	1	2	2	2	1	2	2
7	7.04	3	4	4	7	2	2	1	1	2	2	2	2	1	7	1	4	2	2	1	2	1	4
	6.01	1	2	1	2	2	1	2	4	1	2	1	2	1	2	2	2	2	4	2	2	2	1
	5.01	1	1	1	1	1	4	2	1	1	1	1	1	1	1	1	1	1	1	1	1	1	1
8	7.04	4	4	7	7	7	7	4	*	4	4	5	4	2	7	1	4	2	4	2	4	4	4
	6.01	1	2	1	1	2	4	1	4	2	2	2	4	2	2	2	2	2	2	2	4	2	2
	5.01	2	1	1	7	2	1	2	7	2	2	1	4	2	2	2	2	2	1	2	1	2	2

9	7.04	2	4	1	4	1	1	2	2	2	4	1	7	2	2	2	2	2	4	2	4	2	2
	6.01	1	2	1	4	2	1	1	2	2	2	1	2	2	4	1	2	1	1	1	2	1	1
	5.01	1	1	1	1	2	1	1	2	1	1	1	1	7	4	1	2	1	1	1	1	1	1
10	7.04	4	4	4	7	2	4	4	7	2	2	4	7	2	2	2	2	4	4	3	4	4	4
	6.01	3	2	2	2	4	1	2	7	4	2	2	2	4	1	3	2	3	4	3	4	4	2
	5.01	2	2	2	2	2	2	2	4	3	2	2	4	3	2	2	2	2	2	2	2	2	2
11	7.04	7	*	7	*	4	4	7	7	7	7	7	7	7	*	7	7	7	7	7	7	7	7
	6.01	2	4	2	4	3	1	2	4	2	2	2	4	4	1	2	1	2	2	4	2	3	2
	5.01	2	4	2	4	2	4	2	4	2	7	2	4	2	2	2	2	2	2	2	2	2	2
12	7.04	4	4	4	4	4	4	4	4	1	2	4	4	1	1	2	1	2	2	2	2	2	4
	6.01	2	2	2	2	2	4	2	2	2	1	2	2	2	1	2	1	2	1	2	2	2	2
	5.01	1	1	1	1	1	1	1	1	1	1	1	2	1	1	1	2	1	1	2	1	1	1
13	7.04	7	7	2	4	2	2	2	2	2	2	2	2	2	2	1	2	1	2	1	2	1	2
	6.01	2	1	2	1	2	1	2	1	2	2	2	2	2	2	3	1	2	2	2	1	2	1
	5.01	2	1	1	1	2	1	4	1	2	2	2	2	2	1	1	1	2	1	2	1	2	1
14	7.04	2	7	2	2	4	4	2	4	2	4	2	2	2	2	4	2	2	2	2	2	2	4
	6.01	2	2	1	2	2	1	2	2	2	2	2	2	2	2	2	2	2	2	2	2	2	1
	5.01	1	1	1	1	1	1	1	1	1	4	1	1	2	1	2	1	2	1	1	1	2	1
15	7.04	4	7	4	7	2	2	4	7	4	4	4	4	4	4	4	4	4	4	4	4	4	7
	6.01	2	2	2	4	4	2	2	*	4	4	2	4	4	4	2	4	2	2	3	2	2	1
	5.01	2	1	1	2	2	1	2	4	2	2	3	2	2	2	7	1	2	1	2	2	2	1
16	7.04	2	2	2	2	1	1	2	2	2	4	2	2	2	2	1	2	2	2	2	2	2	2
	6.01	2	4	2	1	2	1	2	2	2	2	2	2	1	2	1	2	1	4	2	2	2	2
	5.01	2	*	2	2	2	1	2	1	2	1	2	2	1	1	1	1	1	1	2	1	2	1
17	7.04	2	2	2	2	**	*	1	4	2	4	2	2	2	2	2	4	1	2	1	2	2	4
	6.01	1	1	1	1	2	2	1	4	2	2	7	4	2	2	2	2	2	2	2	2	4	2
	5.01	1	**	2	1	2	1	1	1	1	1	1	1	1	1	2	1	1	1	2	1	2	1
18	7.04	2	2	2	4	1	1	2	4	2	2	1	2	2	2	2	2	1	2	2	2	2	2
	6.01	1	4	1	2	1	4	1	2	2	2	1	2	3	2	2	2	2	2	2	2	2	1
	5.01	1	4	1	2	1	*	1	2	1	1	1	1	2	4	3	2	1	1	1	1	1	1
19	7.04	4	4	4	4	2	2	2	4	4	4	2	4	4	4	2	4	4	4	4	4	4	4
	6.01	2	7	2	4	2	1	2	4	2	4	2	2	3	2	2	4	2	4	2	4	2	4
	5.01	1	*	1	1	2	4	1	2	2	2	2	2	3	2	1	2	1	2	2	2	1	2

20	7.04	4	4	4	7	4	7	4	7	4	7	7	7	4	7	2	4	4	4	7	7	4	4
	6.01	4	4	4	4	4	7	5	7	7	4	7	4	4	4	4	4	4	4	4	7	4	4
	5.01	3	4	2	4	4	2	4	4	4	4	4	7	3	4	3	7	3	4	4	7	4	4
21	7.04	4	4	7	7	4	4	4	7	7	7	7	7	7	7	7	7	7	7	7	7	7	7
	6.01	2	**	2	2	2	7	2	4	2	2	2	2	1	2	1	2	1	2	1	2	1	2
	5.01	1	**	1	1	1	2	1	1	1	1	1	1	2	1	2	1	1	1	1	1	2	1
22	7.04	2	2	2	2	4	4	2	4	2	4	2	2	2	2	4	4	2	2	2	2	2	2
	6.01	2	*	2	2	2	1	2	2	2	2	2	4	2	1	1	4	1	2	2	2	2	2
	5.01	1	4	1	1	2	1	1	1	2	1	1	1	1	1	1	1	1	1	1	1	1	1
23	7.04	2	7	2	2	1	2	2	2	2	2	2	2	2	2	2	1	3	4	2	4	2	7
	6.01	7	4	5	2	4	1	2	2	4	2	2	2	1	2	1	2	1	2	1	2	1	2
	5.01	2	*	2	1	2	2	2	4	3	1	2	1	2	1	1	1	1	1	1	1	1	1
24	7.04	2	**	1	1	1	1	2	2	2	2	2	2	2	2	1	1	4	4	1	2	1	2
	6.01	2	**	1	2	2	1	1	2	1	2	1	2	2	1	2	1	2	4	2	2	2	1
	5.01	1	**	1	1	2	1	1	1	1	1	1	1	2	1	1	1	1	2	1	1	1	1
25	7.04	2	4	2	2	1	1	2	7	2	2	2	2	2	2	2	2	2	2	2	2	2	2
	6.01	2	*	3	7	4	4	4	2	4	2	4	2	2	1	2	2	2	2	2	1	2	1
	5.01	2	**	2	1	2	4	2	1	2	1	2	1	2	1	2	1	1	1	2	1	2	1
26	7.04	1	2	1	2	2	4	4	7	2	2	2	2	4	4	2	2	2	2	2	2	2	2
	6.01	2	4	2	2	2	7	2	4	2	4	1	2	1	2	1	2	2	2	2	2	1	2
	5.01	2	*	1	1	2	1	2	2	2	2	1	1	1	2	1	1	1	2	1	2	1	1
27	7.04	7	*	2	*	2	*	2	*	4	*	2	*	4	*	4	7	4	7	**	*	2	2
	6.01	1	*	2	2	3	7	2	2	2	2	2	2	2	2	2	4	2	2	2	2	4	2
	5.01	1	**	1	1	2	1	1	1	2	1	1	1	2	2	2	4	2	2	2	2	2	1
28	7.04	1	*	2	4	2	2	4	7	4	4	2	7	4	4	2	4	4	4	4	4	4	4
	6.01	4	2	2	2	4	2	4	4	4	4	2	4	2	2	2	4	2	2	2	4	2	4
	5.01	2	**	2	2	2	7	2	4	2	2	2	2	2	2	2	2	2	2	2	2	2	2
29	7.04	2	*	2	4	2	2	2	7	2	4	2	7	4	4	4	4	4	4	4	4	4	4
	6.01	2	*	2	2	2	2	2	4	3	4	2	4	4	2	4	2	4	2	4	2	4	2
	5.01	2	7	2	2	3	1	3	4	2	2	2	2	3	2	2	2	2	2	2	1	2	2
30	7.04	4	7	2	4	2	2	2	4	2	4	2	4	4	4	4	4	4	4	3	4	4	4
	6.01	2	2	2	2	2	1	2	7	2	7	2	7	4	7	4	4	2	4	4	4	4	2
	5.01	1	*	2	2	2	2	2	2	2	4	2	2	2	2	2	2	2	2	2	2	2	1

31	7.04	2	7	2	7	7	4	2	7	2	*	2	4	4	4	2	4	2	4	2	4	4	4
	6.01	7	2	7	2	7	1	7	2	7	4	7	4	2	2	2	2	2	2	2	2	2	2
	5.01	3	4	3	1	4	7	3	1	4	2	4	2	1	2	1	1	1	2	1	1	1	2

Range identification: **1**:1-100 μg/ml; **2**: 101-200 μg/ml. **3**: 201-300 μg/ml; **4**: 301-400 μg/ml; **5**: 401-500 μg/ml; **6**: 501-600 μg/ml; **7**: 601-700 μg/ml; *: 1250 μg/ml; **: 2500 μg/ml
